# Bridging Levels of Influence: Insights on Policies for Integrating Nurse Practitioners Into Health Systems: Comment on "Development of a Taxonomy of Policy Interventions for Integrating Nurse Practitioners Into Health Systems"

**DOI:** 10.34172/ijhpm.9270

**Published:** 2025-12-14

**Authors:** Efrat Shadmi, Ruth Palan Lopez

**Affiliations:** ^1^The Cheryl Spencer Dapartment of Nursing, The Azrieli Advanced Nursing Center, Faculty of Social Welfare and Health Sciences, University of Haifa, Haifa, Israel.; ^2^William F. Connell School of Nursing, Boston College, Chestnut Hill, MA, USA.

**Keywords:** Advanced Practice Nursing, Nurse Practitioners, Health Workforce, Nurse Practitioner Integration

## Abstract

A taxonomy of policy interventions for integrating nurse practitioners (NPs) into health systems is a valuable tool for addressing implementation barriers across *macro* (policy), *meso* (organizational), and *micro* (clinical) levels. In this commentary we highlight how academic institutions serve as vital conduits between policy, education, and clinical implementation. We explore the role of universities in aligning NP workforce development with system needs through research, interprofessional training, and policy engagement. We highlight *macro–meso* mismatches, particularly when educational capacity outpaces organizational readiness to employ NPs. Advancing NP roles requires coordinated efforts across sectors, and academia, through evidence generation, cross-level engagement, and training innovation, plays a central role in operationalizing the taxonomy and strengthening the contribution of advanced nurses to healthcare systems. Nonetheless, we recognize that academic institutions can at times be inflexible and therefore suggest considering hybrid academic-professional training structures. We conclude with a set of recommendations for research and policy.

## Introduction

 The taxonomy of policy interventions for integrating nurse practitioners (NPs) into health systems, developed by Porat-Dahlerbruch et al,^1^ offers a valuable addition to the growing body of knowledge in this area. By targeting key barriers across three levels: the *macro* (national jurisdiction and regulatory), *meso* (organizational), and *micro* (healthcare team),^2^ the taxonomy provides a structured approach to supporting more effective NP integration into clinical practice.

 This framework is particularly relevant given that the challenges surrounding the implementation of advanced practice nursing in general, and NPs in particular, tend to be consistent across healthcare systems and countries. Common obstacles include resistance from specific stakeholders, particularly within the medical profession. Additional challenges involve regulatory constraints such as outdated or overly restrictive scope-of-practice laws; inadequate financing and reimbursement systems that fail to acknowledge these emerging roles; and limited organizational adoption, often stemming from insufficient leadership support and suboptimal strategies for managing change.^3^ The proposed taxonomy can help bridge the gap by identifying a broad range of policy interventions required for successful integration. These extend well beyond the traditional emphasis, which has mainly centered on establishing state-of-the-art training programs and regulatory frameworks for licensure and authority.

 To advance the taxonomy from concept to implementation, coordinated engagement of key stakeholders is essential, across national, professional, governmental, and organizational levels. The complexity of this integration becomes evident when considering potential disconnects between these levels. For instance, realizing the full potential of advanced nursing roles requires aligning workforce planning with system-level readiness. A foundational requirement for integrating NPs into practice is ensuring an adequate supply of trained and licensed professionals. Educational institutions, typically operating at the macro level, are responsible for preparing the NP workforce. However, when training capacity is not matched by *meso*-level developments, such as organizational infrastructure and resource allocation, mismatches occur: an oversupply of trained NPs may be met with a shortage of available positions. This, in turn, can discourage prospective students from entering NP programs, ultimately worsening workforce shortages.

 Such mismatches have been described in various healthcare settings. For example, in a study of geriatric and palliative care NP programs in Israel, it was found that in the year following NP certification, nearly 40% of geriatric NPs and 60% of palliative care NPs were not employed as NPs.^4^ Similarly, in France, where the NP role was introduced relatively recently, a survey of primary care NPs found that 30% were still working as registered nurses even two years after completing their NP training, primarily due to a lack of available NP positions.^5^ Such gaps may be more prevalent in countries where NP roles have only recently been introduced, compared to those with a more established NP workforce, such as in North America.^3^ Even in the United States, mismatches between workforce preparation and clinical need have been reported. For example, despite a rapidly aging population, U.S. nursing programs have steadily reduced their focus on gerontological NP training, leading to a critical gap between the healthcare needs of older adults and the specialties emphasized in NP education. Only a small fraction of NP programs currently offer gerontology-focused tracks, even as demand for providers with expertise in aging continues to grow.^6^

 These specialty-specific gaps mirror broader concerns about NP surpluses and highlight the persistent need for better alignment between *macro*-level workforce planning and educational pipelines, and the *meso*-level capacity of healthcare organizations to effectively integrate new NP roles. The interconnection between *macro* and *meso* levels is further illustrated through several policy interventions outlined in the taxonomy. For instance, the successful integration of NPs into care teams, and the promotion of interprofessional collaboration, relies heavily on both *macro*-level stakeholder alignment and active engagement from leadership at the *meso* (organizational) level. The historical resistance of the Israel Medical Association to integrating primary care NPs serves as a clear example of how macro-level forces can obstruct reform, a position that is periodically echoed in the opinions voiced by Israeli physicians,^7^ despite local acceptance and support by practicing physicians at the micro level.

## The Role of Academic Institutions

 As central stakeholders in the development and integration of NP roles, academic nursing schools are uniquely positioned to align macro-level policy, meso-level organizational systems, and micro-level clinical practice. Entrusted with advancing health and healthcare, as outlined in the United Nations’ Sustainable Development Goals, and primarily committed to fostering research and education, academia is exceptionally well-placed to play a key role in bridging these levels of influence. The experience of academic institutions in the United States offers a useful case example of how nursing schools can help operationalize this framework across all levels of the system.

 At the *macro* level, nursing schools often engage with national efforts to expand the NP workforce by partnering with policy organizations such as the Future of Nursing: Campaign for Action and the Center to Champion Nursing in America. These groups advocate for removing practice barriers, increasing funding for advanced education, and ensuring that NPs are part of national health workforce planning. Through collaboration with these entities, academic institutions contribute policy-relevant research, shape public discourse, and help align educational priorities with evolving health system needs.

 At the *meso* level, academic institutions serve as strategic partners to healthcare organizations by co-developing and testing models of NP integration. Many universities collaborate with health systems, community clinics, and long-term care providers to create clinical training sites and postgraduate residency programs. These partnerships not only help ensure that NP graduates are prepared for practice in complex settings, but also support smoother organizational integration by building acceptance, role clarity, and workflow alignment. Such models allow academic institutions to respond to workforce and care delivery needs in real time, functioning as a bridge between what is taught and what is needed on the ground.

 At the *micro* level, academic programs influence how NPs are prepared to function in direct patient care roles through the development of competencies in communication, collaboration, and person-centered care. Clinical placements, simulation-based learning, and interprofessional education expose NP students to real-world clinical environments and team-based models of care.^8^ Some programs incorporate simulation and training alongside medical, pharmacy, and social work students to foster collaborative practice from the outset.^9^ These experiences shape how future NPs interact with patients, families, and team members, ultimately influencing the success of NP integration at the point of care. Importantly, such training incorporating interprofessional relationships and clinical team dynamics can influence from the outset how patient-centered care can be enhanced through a collaborative team-based approach.

 Through these multifaceted roles, academia can serve as a vital conduit for supporting the integration of NPs into health systems through several main routes, as outlined below.

## Expansion of the Evidence Base for Nurse Practitioner Integration

 It is widely recognized that translating new knowledge into routine clinical practice can take up to 17 years. Nonetheless, research remains a powerful tool for informing policy and addressing structural barriers. Implementation science can guide policymakers, organizational leaders, and NPs by identifying effective strategies for NP integration and offering insights into how best to apply them in diverse healthcare settings.^10^ To help close the evidence gap and improve understanding of how NPs can operate at their full scope of practice, as well as to examine potential mismatches between NP workforce supply and demand and to develop and evaluate new models for NP integration, research on NP integration should be prioritized by academic institutions and funding bodies. Specifically, research is needed to guide national level policies on how to best balance supply and demand of NPs, especially in light of projected nursing and physician shortages^3^ and to provide a clearer understanding on how to implement NP roles at the full scope of their practice. The NP policy integration taxonomy can guide future research in identifying mechanisms for strengthening integration across macro, meso, and micro levels.

## Expanding Training Beyond Clinical Expertise

 NP training is typically delivered through academic institutions that embed NP curricula within master’s-level nursing programs. This academic setting offers an opportunity to broaden the clinical-professional curriculum to include essential topics such as integration into clinical practice and the development of organizational and management competencies. Indeed, some programs already incorporate these topics,^11^ helping to bridge the gap between *macro*-level education and *meso*-level organizational readiness. They equip NP students with skills for planning their integration into practice and foster strong interpersonal communication competencies.

 Despite the recognized strengths of graduate-level academic education for NPs, in some countries, such as Israel, NP training is currently delivered primarily through non-university, post-master’s certificate programs targeting nurses who already hold a master’s degree, sometimes even in non-nursing fields. Porat-Dahlerbruch and colleagues found that while some physicians acknowledged the potential value of shifting toward academic degree programs, other stakeholders were skeptical, suggesting that such a transition might not meaningfully improve educational quality. Policy-makers also expressed concerns about the increased costs and administrative complexities this move would entail.^1^ Notably, the study did not include perspectives from educators or faculty within academic nursing institutions. Limiting NP education to professional development tracks without integrating it into robust academic frameworks warrants careful re-evaluation. Moreover, the implications of this narrow professional orientation should be considered in the context of the broader levels represented in the policy integration taxonomy. For instance, positioning and legitimizing the NP role may be more challenging when it is perceived as rooted solely in vocational training rather than embedded in an academic and interdisciplinary context.

 Nonetheless, universities’ inherent rigidity can impede efforts to align healthcare workforce demands with available training capacity, as inflexibility, interfaculty silos, and funding limitations frequently constrain their responsiveness. Therefore, new solutions, such as academic nursing institutions partnering with professional associations, can generate hybrid models accounting for the benefits and shortcomings of each modality. Hybrid academic-professional models can provide the academic rigor and backbone, through incorporation of courses such as evidence-based nursing, theoretical and critical thinking, as well as the foundation for research methods. Also, they can incorporate the highly professional clinical specialty training required for producing the highest quality NP workforce in settings best fitted for such training, embedded within healthcare organizations. Such models have been recently introduced in some of the academic nursing institutions in Israel and their implementation should be duly evaluated.

## Generating Interprofessional Training and Practice Models

 A well-recognized challenge in health professions education is its tendency to operate within disciplinary silos, with limited opportunities for interprofessional learning. This stands in stark contrast to the widely accepted understanding that high-quality, team-based care depends on interprofessional education, learning with, from, and about other professions.^12^ Interprofessional education enhances students’ understanding of both their own and others’ professional roles. This fosters mutual respect and collaboration, which in turn can improve care quality, ultimately benefiting the patient through more integrated and comprehensive care.^13^ However, research on how best to design and implement effective interprofessional training for NPs remains limited, with most existing studies narrow in scope and context.^14^

 Academic institutions are uniquely positioned to help address this *macro*–*meso*–*micro* divide, as they serve as environments where multiple disciplines converge in both education and research. Unlike professional training hubs, universities offer the structural and intellectual infrastructure needed to foster interprofessional collaboration and innovation. Indeed, studies that examine the impact of interprofessional NP and physician post-graduate programs signify its benefits.^15^

 Nevertheless, interprofessional education is sparse. Developing theoretically grounded and empirically tested interprofessional NP education is essential for fostering stakeholder collaboration at the macro level. Such education can support the formalization of NP role definitions and competencies within regulatory frameworks and practices endorsed by national physician and health profession bodies. At the meso level, interprofessional education is equally critical, as it can serve as the foundation for facilitating physician leadership engagement, fostering mutual understanding of the NP role, and providing physicians and other health professionals with early exposure to NP practice, well before integration into clinical units or teams. At the micro level, it can serve as a strong basis for demonstrating the value of NPs to the healthcare team and serve to break silos and improve team communication.

## Conclusion

 Despite variation in how NP roles are defined, adopted, and operationalized across countries, there is a shared imperative: to identify and implement effective strategies that enable the full integration of this valuable workforce into healthcare systems. The taxonomy of policy interventions developed by Porat-Dahlerbruch and colleagues offers a solid foundation for guiding these efforts across *macro* (policy and regulation), *meso* (organizational structures), and *micro* (clinical teams) levels. To move from framework to implementation, bridging mechanisms that support coordination across these levels are essential. Academic institutions, alongside other key stakeholders such as policymakers, professional associations, and healthcare organizations, can contribute meaningfully to this process. To achieve these goals, clear and structured curricula fostering interprofessional education and incorporating implementation strategies, should be mandated to ensure that NPs possess skills required for their effective integration into practice. Moreover, governing bodies should engage all key stakeholders, including academic leaders, professional representatives, nurses, physicians, other health professionals, and organizational managers, in shaping training programs and role-placement guidelines to reduce barriers to effective NP implementation.

 Thus, through education, research, interprofessional engagement, and strong collaborative structure, academia is well-positioned to support integration efforts. Ultimately, maximizing the impact of NP roles on health systems and population health requires cross-level alignment and sustained cooperation among all relevant actors.

## Disclosure of artificial intelligence (AI) use

 Not applicable.

## Ethical issues

 Not applicable.

## Conflicts of interest

 Authors declare that they have no conflicts of interest.
